# The effect of robot-navigation-assisted core decompression on early stage osteonecrosis of the femoral head

**DOI:** 10.1186/s13018-019-1437-x

**Published:** 2019-11-21

**Authors:** Benjun Bi, Shudong Zhang, Yuchi Zhao

**Affiliations:** 1 Orthopaedic Department, The Affiliated Hospital of Qingdao University, Wutaishan Road No. 1677, Huangdao District, Qingdao, Shandong Province China; 2 Department of Orthopaedic Surgery, Yantaishan Hospital, Jiefang Road No. 91, Yantai, Shandong Province China; 3 Department of Osteoarthropathy, Yantaishan Hospital, No. 91, Jiefang Road, Yantai, 264001 Shandong Province China

**Keywords:** Avascular necrosis of the femoral head, Robot navigation, Core decompression

## Abstract

**Background:**

The aim of the current paper is to evaluate the effects of robot-navigation-assisted core decompression compared with conventional core decompression surgery for early-stage osteonecrosis of the femoral head.

**Methods:**

Twenty patients with a total of 36 hips who were diagnosed with Association Research Circulation Osseous stage 2 avascular necrosis of the femoral head and who received core decompression with or without robotic assistance were reviewed. The Harris hip score and visual analog scale score were used to assess clinical function. Intraoperative radiation exposure and operation time were used to evaluate the effectiveness of the robot-assisted system.

**Results:**

At a mean follow-up of 26.4 months (24–36 months), the Harris hip score, visual analog scale score, and survival rate of the patients were similar between the conventional and robot-assisted groups. The guidewire insertion time, number of guidewire attempts, and radiation exposure during guidewire insertion were all significantly lower in the robot-assisted group than in the conventional group.

**Conclusions:**

Robot-assisted core decompression of the femoral head is as safe and effective as a conventional core decompression surgery. It can reduce operation time and decrease intraoperative radiation exposure.

## Background

Osteonecrosis of the femoral head (ONFH) is one of the main reasons for total hip arthroplasty (THA) in young patients [1]. This pathology accounts for 5% to 46.9% of THA undertaken in younger age groups [2, 3]. A major disadvantage of THA is that after a mean period of 15 to 25 years, most implants become loose and require revision surgery [4]. Therefore, hip-preserving options for treating ONFH, apart from THA, are needed. The most commonly chosen surgical treatment option is core decompression, which is performed by drilling into the necrotic lesion to release pressure in the affected tissue and to encourage ingrowth of new blood vessels [5–7]. Different variations of the conventional core decompression technique have been described, e.g., multiple drilling or core decompression in combination with bone marrow mononuclear cells and vascularized fibular or cancellous bone grafts [8–18]. The conventional freehand fluoroscopy technique is very common for intraoperative visualization achieved via an image intensifier and requires complex hand-eye coordination [19–21]. Recently, computer-assisted orthopedic surgery, which potentially increases the accuracy and efficiency of percutaneous targeting, has utilized image navigation systems and purpose-built robots. These techniques reduce the time of repeated C-arm movements during surgery and optimize the precise and simultaneous visualization of the surgical instruments in relation to the patient’s anatomy [22]. However, navigation does not solve the problem of precise manipulation for guidewire insertion and does not calculate the instrument trajectory. Robot-assisted orthopedic surgery is believed to potentially improve the precision of implant placement and decrease radiation and operative time [23]. The research of several manufacturers has resulted in the production of hardware and software products for orthopedic surgery [24]. TiRobot™ is an orthopedic surgery robot that can be used for the implantation of different guidewires and screws and is especially useful for guidewire insertion of the proximal femur. The method, which has been studied in cadaver and cohort studies, has shown high accuracy. To date, there has been no report describing a direct comparison of robot assistance with the conventional freehand technique. This study was designed to evaluate the accuracy of robot-assisted core decompression (RCD) combined with cancellous bone grafts compared with freehand conventional core decompression (CCD) for the treatment of early-stage osteonecrosis of the femoral head.

## Methods

The present study was conducted as a case-controlled, retrospective study. Patient privacy and data confidentiality were maintained throughout the research process. A full ethical review and then approval from the Institutional Review Board were obtained prior to the commencement of the study.

### Patient selection

The study reviewed 20 patients who had undergone 36 hip-preserving surgeries to treat ONFH for defects classified as stage 2 according to the ARCO (Association Research Circulation Osseous) classification between January 2016 and December 2017 in the Orthopedic Department of Yantaishan Hospital. The diagnosis of non-traumatic hip osteonecrosis was based on the radiological analysis and clinical history. Our inclusion criteria involved previously unoperated hips, where preoperative plain radiographs and MRI scans were available for review. The presence of bandlike abnormal signals, bandlike hypointense zones on T1-weighted images, and matching hyperintense zones on short tau inversion were used as key findings to diagnose hip ON on MRI scans. The exclusion criteria were classifications of ARCO stages 3 and 4, surgical contraindications, having received any other type of surgical treatment, and secondary arthritis. Patients whose clinical records and serial radiographs were not complete and available were also excluded. Patients willingly received core decompression of the femoral head with or without the use of a robot navigation system. Nine patients with a total of 16 hips were enrolled in the RCD group, and 11 patients with a total of 20 hips were enrolled in the CCD group. Radiographs with anteroposterior and lateral views of the pelvis and both hips as well as magnetic resonance imaging (MRI) scans were available to be reviewed by one experienced radiologist to evaluate the degree of ONFH preoperatively. All operations were performed by the second author under general anesthesia.

### Conventional freehand core decompression

**Fig. 1:**
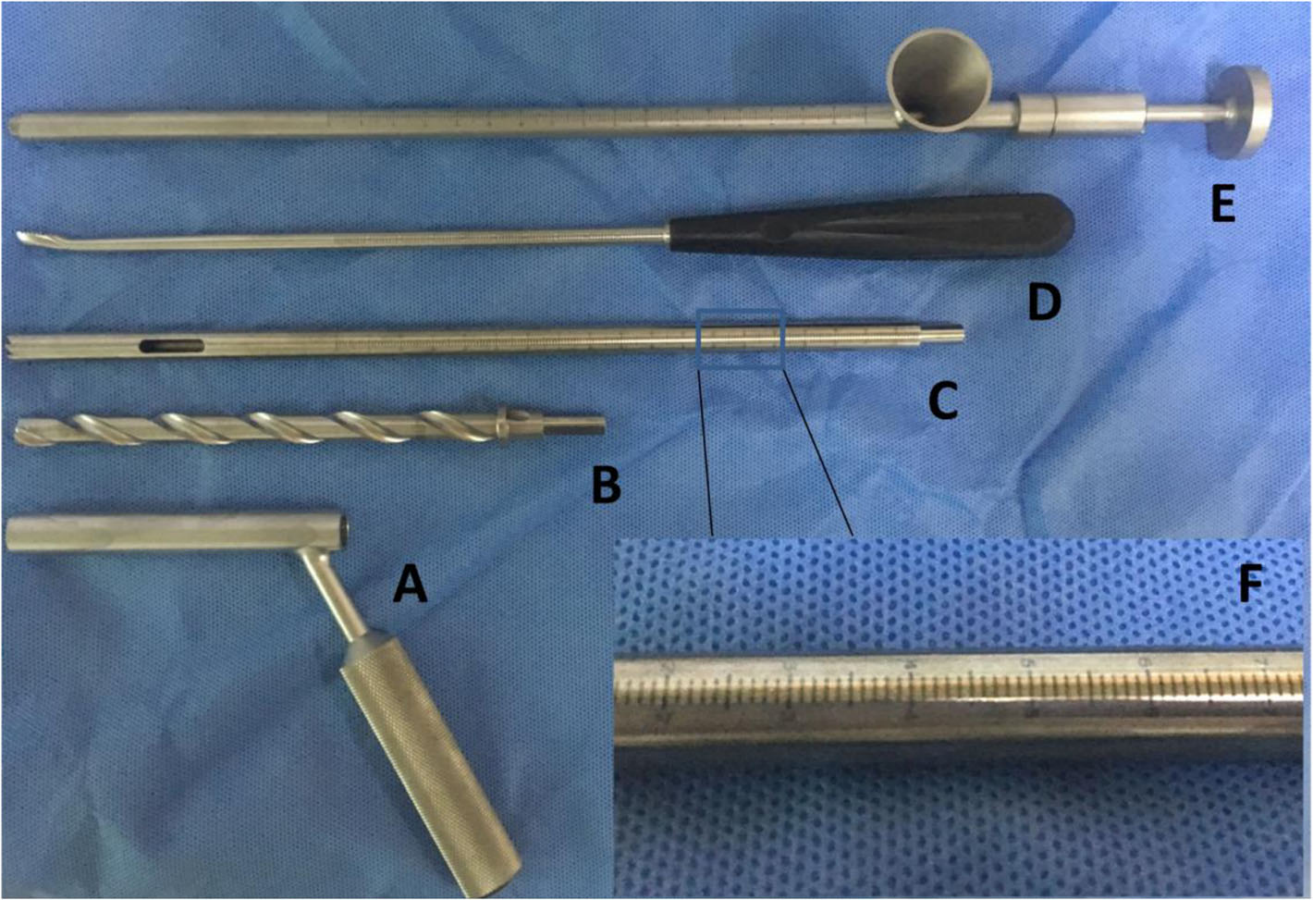
Surgical instruments used for core decompression and bone graft of the femoral head. **a** Soft tissue protecting sleeve with an inner diameter of 10 mm. **b** Solid trephine used to penetrate to lateral cortex of the femur and make a short tunnel within the proximal femur. **c, f** Hollow trephine with a diameter of 10 mm used for core decompression of the necrotic area. The scale on the hollow trephine is used for monitoring the location of the trephine in the femoral head with an accuracy of 1 mm. **d** Long curette used for removing necrotic bone of the femoral head. **e** Grafting pipe with a funnel at one end through which cancellous bone can be easily put into the pipe and then impacted into the femoral head through the long pipe

All procedures were carried out in the supine position using an orthopedic table with traction guided by an image intensifier. A direct lateral approach of the hip joint was utilized in the CCD group. A guidewire was then drilled into the necrotic area, and a hollow trephine of 10 mm in diameter was introduced into the neck of the femur from an opening in the greater trochanter; it passed towards and into the head of the femur, stopping 5 mm short of the articular cartilage along the guidewire. This introduction has been shown to be controlled in both the anteroposterior and lateral planes with an image intensifier [6]. Curettes were used to remove necrotic bone from the anterosuperior aspect of the femoral head. Then, the cancellous bone harvested from the femoral head and neck was reinserted into the core using a grafting pipe (Fig. 1).

### Robot component

**Fig. 2:**
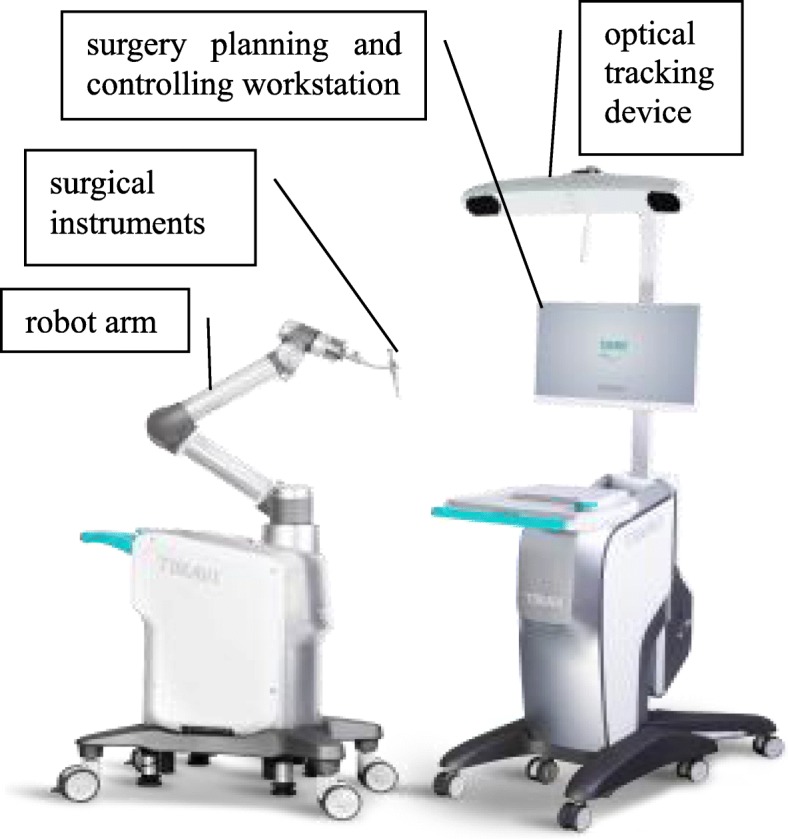
Main components of TiRobot™: a robot arm, an optical tracking device, a surgical planning and controlling workstation, and some surgical instruments

**Fig. 3:**
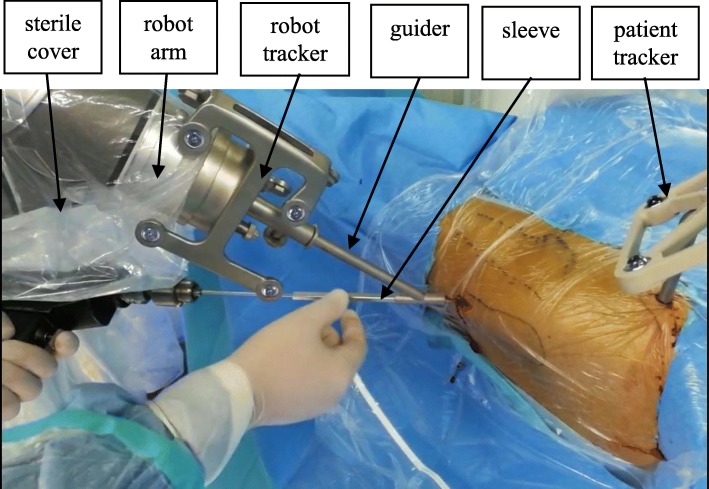
Surgical instruments: robot arm is isolated by the sterile cover, and robot tracker and patient tracker are fixed, respectively, at the distal end of the robot arm and on the patient, guider attaches to the robot arm and firmly holds the sleeve; the sleeve can slide along the guider and invade patient

For patients enrolled in the RCD group, an orthopedic surgery robot, TiRobot™ (TINAVI Medical Technologies Co., Ltd., Beijing, China), was used. This robot system consisted of a robot arm, an optical tracking device, a workstation for surgical planning and control (Fig. 2), and surgical instruments (Fig. 3). The robot arm was an actuator for planning the trajectory in this system with 6 degrees of freedom. The optical tracking device was a binocular camera based on infrared light, whose positioning error was <0.3 mm. The robot tracker and the patient tracker with reflection balls were fixed. The optical tracking device was used to locate the spatial position of the robot arm and the patient through the robot tracker and patient tracker, respectively. The calibrator was used to acquire the mapping relation between the imaging space and the surgical space through the matching coordinates of fluoroscopic images and calibrator images, respectively. The workstation used for planning and control was used for image processing and trajectory planning as well as calculating the coordinates, saving the data and controlling the robot arm movements.

### Robot-assisted core decompression

After preparing the position, sterilizing, and draping, the patient tracker was fixed on the ipsilateral anterior superior iliac spine of the surgical site, and a C-arm was placed on the same side of the patient. The robot tracker and calibrator were assembled at the distal end of the robot arm. After the calibrator was fixed, anteroposterior and lateral intraoperative fluoroscopic images were taken. The fluoroscopic images were automatically imported into the workstation for planning and control. Based on these fluoroscopic images, the surgeon planned the surgical trajectory for guidewire insertion and generated spatial positioning orders for the robot arm. The arm was moved automatically according to the orders from the surgical workstation for planning and control, and the position of the surgical trajectory was completed.

**Fig. 4:**
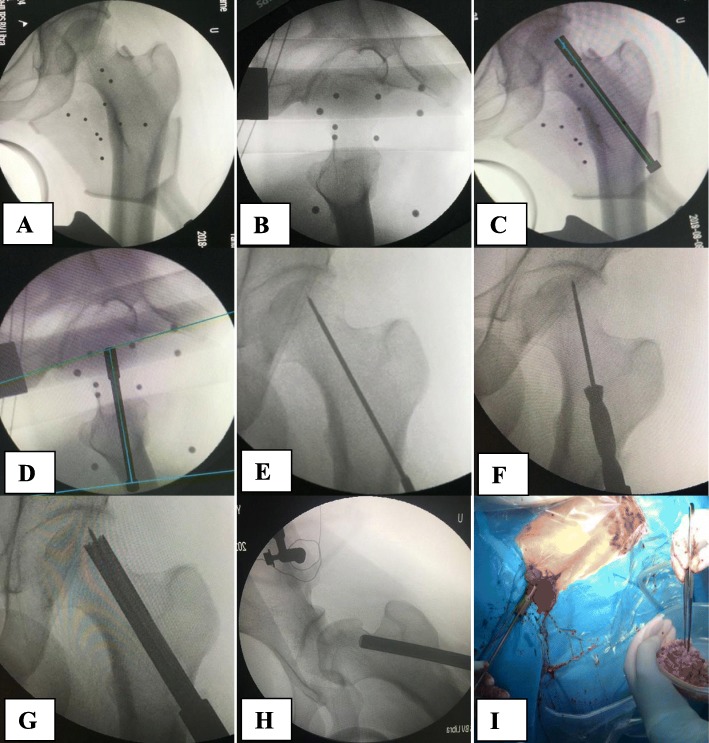
Illustration of the robot-assisted core decompression and bone graft. **a, b** Anteroposterior and lateral intraoperative fluoroscopic images of the hip were taken and input to the workstation. **c, d** Surgical trajectory for guidewire insertion on anteroposterior and lateral view of the hip. **e** Guide wire insertion. **f** Solid trephine insertion along the guidewire. **g** Hollow trephine insertion along the guidewire into the necrotic area of the femoral head. **h**, **i** Grafting of the cancellous bone using the grafting pipe with a funnel

During the positioning process, the surgeon controlled the accuracy by adjusting the guidewire trajectory on the fluoroscopic image, as necessary. When the positioning accuracy was <1.00 mm, the guidewire was placed into the sleeve. The instrumentation was then concluded through decompression and bone grafting over the guidewire without the assistance of the robot (Fig. 4). The surgeon verified the accuracy through a comparison between the position of the inserted guidewire and the planned position intraoperatively through fluoroscopy.

### Postoperative management and clinical evaluation

Postoperatively, a standard rehabilitation protocol was utilized in all patients. Patients in both groups were kept non-weight-bearing for 6 weeks followed by partial weight-bearing for the subsequent 6 weeks using a crutch in the opposite hand. They began full weight-bearing as tolerated 3 months postoperatively. Data were collected retrospectively with the Harris hip score (HHS) and visual analog scale (VAS) score obtained preoperatively and at the last follow-up. The mean follow-up time was 26.4 months (24–36 months). The survival rates of the femoral head were compared between the groups. We defined surgical failure as the need for hip replacement surgery or radiographic changes together with lesion progression during the postoperative follow-up compared to the preoperative data.

### Evaluation of the efficacy of the robot-assisted system

To evaluate the efficacy of the robot-assisted system, the duration of trajectory planning, the duration of surgery after making the skin incision, the insertion time of the guidewire during surgery, the number of guidewire attempts made during surgery, the radiation exposure until the insertion of the guidewire, and the radiation exposure between the completion of guidewire insertion and skin closure were assessed.

All statistical analyses were performed with SPSS version 20.0 (Chicago, IL, USA). Patient age, BMI, the pre- and postoperative HHS and VAS score, operation time, guidewire insertion time, and intraoperative radiation exposure time were compared using Student’s *t* test. Additionally, Fisher’s exact probability test was used to analyze sex, ARCO classification, and survival rate of the patients. A 5% significance level was applied for all tests (*p* < 0.05).

## Results

Twenty patients with a total of 36 hips were enrolled in this study: 9 patients with a total of 16 hips in the RCD group and 11 patients with a total of 20 hips in the CCD group. Both groups were similar in terms of age, sex, body mass index (BMI), and ARCO classification (Table 1). No intraoperative or postoperative complications occurred in any patient. The mean follow-up time was 26.8 ± 3.42 months in the RCD group and 26.4 ± 3.65 months in the CCD group (*p* = 0.770).

**Table 1:** Clinical characteristics and results

	CCD group (*n* = 20 hips)	RCD group (*n* = 16 hips)	*p*
Age (years)	35.30 ± 4.72	34.88 ± 3.81	0.772
Gender			1.000
Male	8	7	–
Female	3	2	–
BMI (kg/m^2^)	26.32 ± 3.50	26.45 ± 2.99	0.903
ARCO classification			0.648
2a	3	1	–
2b	4	2	–
2c	13	13	–
HHS (preoperative)	68.95 ± 4.80	69.38 ± 3.65	0.771
VAS (preoperative)	4.10 ± 0.91	3.75 ± 0.93	0.265
HHS (last follow-up)	86.00 ± 4.29	86.00 ± 3.72	1.000
VAS (last follow-up)	1.40 ± 0.883	1.31 ± 0.704	0.749

The preoperative HHS and VAS scores were similar between the two groups (Table 1). The HHS and VAS scores at the last follow-up were also similar between the two groups. The mean HHS and VAS score of all patients increased significantly from 69.14 ± 4.27 points and 3.94 ± 0.92 points preoperatively to 86.00 ± 3.99 points and 1.36 ± 0.80 points at the last follow-up (*p* < 0.001 and *p* < 0.001, respectively).

**Fig. 5:**
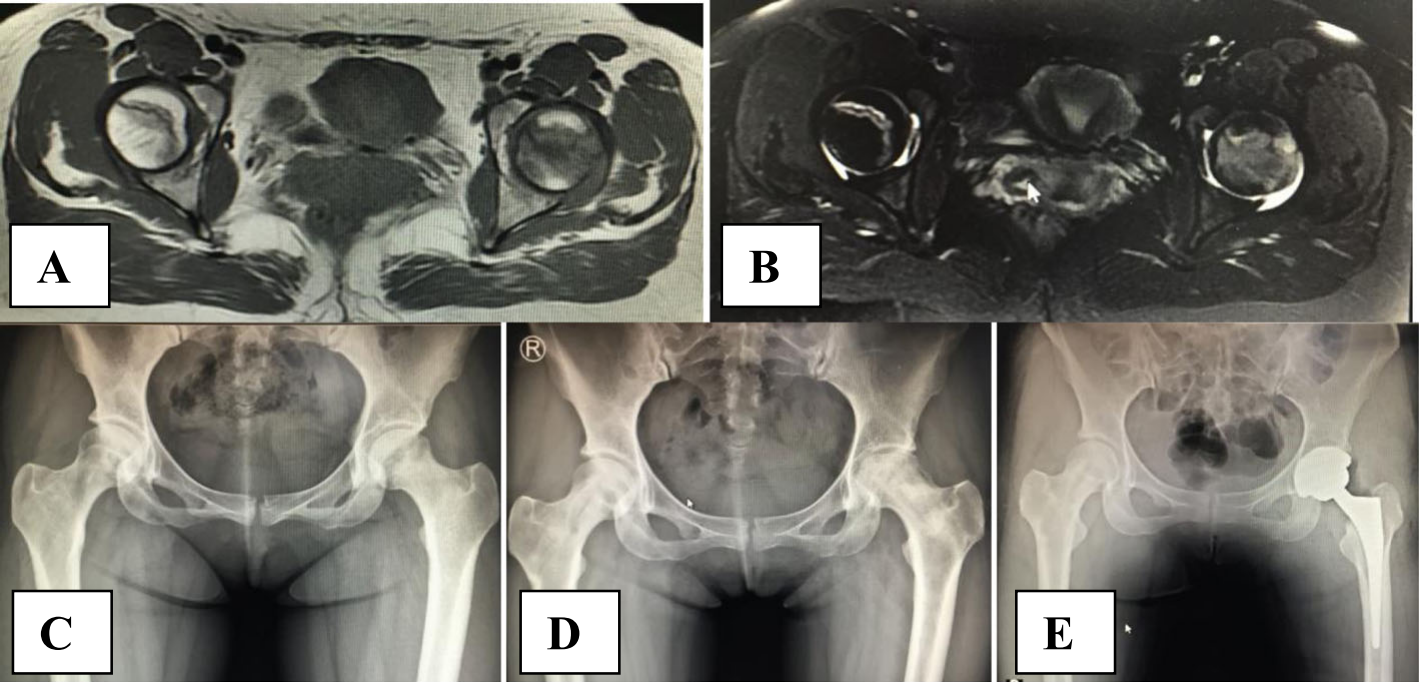
Clinical case presentation. A 29-year-old woman, bilateral hip pain for 3 months with glucocorticoid use for 3 years before hospital admission. MRI T1WI (**a**), T2WI (**b**), and anteroposterior X-ray film (**c**) showed stage 2c osteonecrosis of two hips according to the ARCO classification. Two years after robot-assisted core decompression of two femoral heads, X-ray film showed the collapse of the left femoral head (**d**) with the right side intact. Finally, the patient received a total hip replacement of the left hip joint (**e**)

The total survival rate in this study was 72.2%. According to the definition of failure, at the last follow-up, four hips in the RCD group and 6 hips in the CCD group failed, and the patients received total hip replacement surgery with survival rates of 75.0% and 70.0%, respectively (*p* = 1.000). The 10 hips that failed and thus required total hip replacement surgery (Fig. 5) were all classified as ARCO stage 2c preoperatively (Table 2).

**Table 2:** Results of the survival rate according to ARCO stage

Stage	Survival rate		*p*
	CCD group	RCD group	
2a	3/3	1/1	–
2b	4/4	2/2	–
2c	7/13	9/13	–
2 (all)	14/20	12/16	1.000

The duration of intraoperative planning of the guidewire trajectory using TiRobot™ software was 2.38 ± 0.50 min in the robot-assisted group, whereas no planning time was necessary for the freehand group (Table 3). The operation time after making the skin incision was significantly longer in the CCD group than in the RCD group (*p* < 0.001). Even after combining the planning time and operation time together as the total operation time, the operation time was still significantly longer in the CCD group than in the robot-assisted group (60.15 ± 8.42 min vs. 38.25 ± 4.14 min, *p* < 0.001). The surgical time for guidewire implantation was significantly shorter in the robot-assisted group than in the conventional freehand group (2.38 ± 1.36 min vs. 17.25 ± 6.18 min, *p* < 0.001). The number of guidewire attempts was significantly different between the CCD group and the RCD group (6.40 ± 2.60 times vs. 3.19 ± 1.76 times, *p* < 0.001). The radiation exposure for guidewire insertion was also significantly different between the CCD group and the RCD group (6.95 ± 2.09 s vs. 3.19 ± 1.28 s, *p* < 0.001). The radiation exposure after guidewire insertion was similar between the CCD group and the RCD group (7.65 ± 1.04 s vs. 7.31 ± 1.01 s, *p* = 0.335).

**Table 3:** Evaluation of the efficacy of the robot-assisted system

	CCD group (*n* = 20 hips)	RCD group (*n* = 16 hips)	*T*	*p*
Duration of trajectory planning (min)	Not applicable	2.38 ± 0.50	–	–
Duration of surgery (min)	60.15 ± 8.42	35.88 ± 3.83	10.663	< 0.001
Guide wire insertion time (min)	17.25 ± 6.18	2.38 ± 1.36	9.421	< 0.001
Number of guidewire attempts	6.40 ± 2.60	3.19 ± 1.76	4.219	< 0.001
Radiation exposure for guide wire insertion (s)	6.95 ± 2.09	3.19 ± 1.28	6.312	< 0.001
Radiation exposure after guide wire insertion (s)	7.65 ± 1.04	7.31 ± 1.01	0.978	0.335

## Discussion

Core decompression of the hip is the most common procedure used to treat early stages of ONFH [6]. Different variations of the conventional core decompression technique have been described, e.g., multiple drilling or core decompression in combination with bone marrow mononuclear cells and vascularized fibular or cancellous bone grafts [8, 9], to enhance bone repair. However, a systematic review of the literature showed that, at present, there is no one treatment that is superior to the others in terms of the treatment of ONFH [13]. After 30 months of follow-up, patients treated with the advanced core decompression technique without autologous bone grafts showed a hip survival rate of 67%, which was nearly the same as the survival rate of 65% reported for conventional core decompression [25, 26]. The improvement demonstrated in our study may be explained by the better biomechanical properties of autologous cancellous bone in comparison to other bone graft substitutes. Compared with artificially synthesized bone graft substitutes and bone marrow aspirate from the iliac crest, autologous bone is osteoconductive, osteoinductive, and osteogenetic, resulting in good bone remodeling [11–14]. Vascularized fibular grafts, which require a complex and labor-intensive procedure that involves microsurgical anastomosis of the vessels [15, 16], are widely used. Because of the complexity of free vascularized fibular graft surgery and to perform this procedure efficiently, the technique is often performed using two teams of surgeons working simultaneously. For that reason, most of these surgeons continue to perform core decompression today [17, 18].

Lesion sizes in the femoral head have been found to be a very important factor in influencing progression. There were lower failure rates found when the lesion involved less than 15% of the femoral head or had a necrotic angle of less than 200° (14–25%) and when the osteonecrotic lesion involved only the medial one-third of the weight-bearing surface [27]. Ten hips failed and required total hip replacement surgery in this study. Because of the limited diameter of the decompression trephine of 10 mm, the amount of remaining necrotic tissue after the procedure was still substantial for those patients classified as ARCO stage 2c. The outcome of core decompression is reported to be good for small necrotic defects. For patients with larger defects, an increasing number of surgeons now attempt to remove as much necrotic tissue as possible, as this procedure yields much better results [10, 28]. The amount of preoperative and remaining necrosis correlates significantly with treatment failure. The larger both volumes are, the more likely it is that treatment will fail. In patients with remaining necrosis of less than 1000 mm^3^, no treatment failure is observed [29].

In addition, accurate decompression of the necrotic area of the femoral head indicates treatment success. The conventional freehand fluoroscopy technique is very common for intraoperative visualization achieved via an image intensifier, which is possible in only one plane at a time while requiring complex hand-eye coordination [20, 21]. Statistically, the rates of malposition for the use of guidewires under fluoroscopic guidance have been reported to range from 2 to 15% [30, 31]. Intraoperative computer-assisted techniques and computer tomography-based techniques have been introduced to improve accuracy and decrease radiation exposure during surgery [22]. However, these techniques do not solve the problem of the precise manipulation for guidewire insertion and do not calculate the instrument trajectory [23]. Robot-assisted orthopedic surgery is believed to potentially improve the precision of implant placement and decrease radiation and operative time [23, 24, 32]. TiRobot™ is an orthopedic surgery robot that can be used for the implantation of different guidewires and screws and is especially useful for guidewire insertion of the proximal femur and spine. This robot-assisted surgery system was first introduced in spinal surgery for pedicle screw insertion by Wei Tian [23, 33], and it was suggested that the accuracy of the robot-assisted technique was superior to that of the freehand technique. In this study, the number of guidewire attempts and the guidewire insertion time was both significantly improved with robotic assistance, resulting in decreased intraoperative radiation exposure. The overall operation time was shorter in the robot-assisted group than in the conventional group, mainly due to the faster insertion of the guidewire. When the guidewire insertion time was adjusted, the remaining operation time and radiation exposure were similar between the two groups.

TiRobot™ was created for orthopedic surgery based on the use of an intraoperative C-arm and combines navigation and robot techniques to enable accurate positioning, adequate steadiness, and repeatability [34]. The most accurate aspect of TiRobot™ is derived from the electro-optical camera and the robot arm based on a specific biplane orientation algorithm in order to achieve a high-precision and stable operation. The novel biplane algorithm can calculate the guidewire trajectory, depending on two intraoperative fluoroscopy images. According to the calculated surgical trajectory, the robot arm moves automatically to the correct position and maintains a precise and steady sleeve. Drilling and guidewire insertion are still performed manually by the surgeon. It is not necessary to repeatedly verify the surgical trajectory using intraoperative fluoroscopy. The high precision of the specific biplane orientation software based on two intraoperative fluoroscopy images can explain the reduction of radiation exposure in the robot-assisted group [23].

Robot-assisted core decompression is believed to potentially improve the precision of surgical procedures. It can reduce the operation time and decrease the intraoperative radiation exposure compared with conventional free-hand surgery. In addition, this procedure is minimally invasive due to a small incision and has a soft tissue protecting sleeve, thus allowing for a fast recovery and a short hospital stay. However, additional time is required for the intraoperative planning of the guidewire trajectory. Finally, the use of the robot-assisted system results in high medical expenses.

There are some limitations to this study. First, the sample size is small. It is difficult to compare hips with ARCO stages 2a, 2b, and 2c separately. Second, the duration of follow-up is short and may result in overestimating the long-term survival rate. Third, the study also includes a control group, and factors that may influence the progression are not matched. Further rigorous randomized controlled trials are needed for more convincing results.

## Conclusions

Robot-assisted core decompression of the femoral head is as safe and effective as a conventional core decompression surgery. It can reduce operation time and decrease intraoperative radiation exposure.

## Data Availability

The datasets used during the current study are available from the corresponding author upon reasonable request.

## Abbreviations

ARCO: Association Research Circulation Osseous
BMI: Body mass index
CCD: Conventional core decompression
HHS: Harris hip score
ONFH: Osteonecrosis of the femoral head
RCD: Robot-assisted core decompression
THA: Total hip arthroplasty
VAS: Visual analog scale
